# HIV risk among young women who sell sex by whether they identify as sex workers: analysis of respondent‐driven sampling surveys, Zimbabwe, 2017

**DOI:** 10.1002/jia2.25410

**Published:** 2019-12-03

**Authors:** Bernadette Hensen, Sungai T Chabata, Sian Floyd, Tarisai Chiyaka, Phillis Mushati, Joanna Busza, Isolde Birdthistle, James R Hargreaves, Frances M Cowan

**Affiliations:** ^1^ Faculty of Infectious and Tropical Diseases London School of Hygiene and Tropical Medicine London United Kingdom; ^2^ Centre for Sexual Health and HIV/AIDS Research (CeSHHAR) Zimbabwe Harare Zimbabwe; ^3^ Faculty of Epidemiology and Population Health London School of Hygiene and Tropical Medicine London United Kingdom; ^4^ Faculty of Public Health and Policy London School of Hygiene and Tropical Medicine London United Kingdom; ^5^ Department of International Public Health Liverpool School of Tropical Medicine Liverpool United Kingdom

**Keywords:** female sex worker, adolescents and young women, HIV infection, HIV prevention, Zimbabwe, Sub‐Saharan Africa

## Abstract

**Introduction:**

Across sub‐Saharan Africa, selling sex puts young women at high risk of HIV. Some young women who sell sex (YWSS) may self‐identify as sex workers, while others may not, having implications for how to reach them with HIV prevention. We describe characteristics, sexual behaviours and health service use of YWSS in Zimbabwe, comparing women who identified as female sex workers (FSW) and women who did not (non‐identifying‐YWSS), and explore factors associated with HIV infection.

**Methods:**

We analysed data from respondent‐driven sampling (RDS) surveys among YWSS aged 18 to 24 implemented in six sites in Zimbabwe from April to July 2017. RDS was used to enrol YWSS into an impact evaluation of the multi‐country DREAMS (Determined, Resilient, Empowered, AIDS‐free, Mentored and Safe) Partnership, which provides comprehensive HIV prevention programming to adolescent girls and young women. Women completed an interviewer‐administered questionnaire and were offered HIV testing services. We used logistic regression (RDS‐II‐weighted, normalized by site) to identify factors associated with prevalent HIV infection.

**Results:**

Forty‐four seeds recruited 2387 YWSS. RDS‐adjusted HIV prevalence was 24%; 67% of women identified as FSW. FSW were older and had lower educational attainment than non‐identifying‐YWSS. While 40% of FSW reported 10+ clients in the previous month, 9% of non‐identifying‐YWSS did so. FSW were more likely to have accessed HIV‐related services, including HIV testing in the last six months (FSW: 70%; non‐identifying‐YWSS: 60%). Over half of all YWSS described selling sex as their main financial support (FSW: 88%; non‐identifying YWSS: 54%). Increasing age, lower educational attainment, younger age of first selling sex and higher number of clients in the previous month were associated with prevalent HIV.

**Conclusions:**

YWSS in Zimbabwe have a high prevalence of HIV, reported high numbers of sexual partners and depend financially on selling sex. Non‐identifying‐YWSS differed socio‐demographically to FSW, yet factors associated with HIV risk were similar for all women. Women not identifying as FSW were less likely to access services, suggesting they should be prioritized for HIV prevention. Network‐based recruitment may enhance their inclusion in programmes, like DREAMS, which aim to reach young women at highest‐risk with comprehensive health, HIV prevention and social protection services.

## 1 Introduction

In sub‐Saharan Africa, adolescent girls and young women aged 15 to 24 are more vulnerable to HIV infection than their male peers and older women 1. At particularly high risk are young women who sell sex (YWSS) 2, 3. We define YWSS as women aged <25 years who consider themselves engaged in sex work, and may self‐identify as female sex workers (FSW) or not. Evidence suggests that YWSS are less able to negotiate condom use than older FSW, are more likely to have age‐disparate sexual partnerships, and have less access to available HIV prevention and other health services for fear of stigma and discrimination from healthcare workers 2, 4, 5, 6, 7, 8 The illegality of selling sex exacerbates vulnerabilities of YWSS and puts them at high risk of HIV 7.

Over 50% of self‐reported, adult FSW in Zimbabwe are estimated to live with HIV 9. A cohort analysis of FSW attending a national FSW HIV programme estimated that annual HIV incidence among FSW aged <25 was 11% between 2009 and 2013 10. Experience from this programme has suggested that there may be a sizeable population of YWSS who are under‐represented in surveys and not well‐served by services targeted at FSW 2, 10, 11, 12. Some YWSS may not be formally engaged in sex work while others sell sex occasionally. Unlike transactional sex, which has been associated with implicit exchanges within romantic relationships, and with agency and power 13, 14, this period of selling sex is likely, in part, driven by economic need 15. YWSS may later begin to identify as FSW and only then recognize the period before this transition as sex work. During this transition, YWSS may be particularly vulnerable to HIV, being harder to reach with programmes and less supported and skilled in, for example, negotiating condom use with clients. However, there are little empirical data on younger women who sell sex. A better understanding of the HIV risk of YWSS and whether and how risk differs between YWSS who consider themselves formally or not formally engaged in sex work would support developing programmes that meet the needs of these different women 16.

The DREAMS (Determined, Resilient, Empowered, AIDS‐free, Mentored and Safe) Partnership seeks to deliver a package of evidence‐based HIV prevention interventions, including clinical services and social protection interventions, to adolescent girls and young women, including YWSS, in ten sub‐Saharan African countries 17. In Zimbabwe, DREAMS includes an offer of oral pre‐exposure prophylaxis (PrEP) to young women at highest risk of HIV, including YWSS. Recognising that there are a sizeable number of YWSS, we used a network‐based recruitment strategy to reach YWSS, including women who do not identify as FSW, as well as women who do, and enrolled these women in a cohort study as part of an impact evaluation of DREAMS. This evaluation of the impact of DREAMS on HIV incidence among YWSS aged 18 to 24 is ongoing in six sites in Zimbabwe 18. In this analysis, we used data collected at enrolment to describe HIV prevalence, sociodemographic characteristics, risk behaviours and use of services among YWSS. We investigated whether these factors differed between YWSS who self‐identified as FSW and those who did not, and investigated risk factors associated with prevalent HIV infection.

## 2 Methods

### 2.1 Study setting

From April to July 2017, we conducted respondent‐driven sampling (RDS) surveys in six sites across Zimbabwe, two sites where DREAMS was implemented and four non‐DREAMS sites selected for comparison. RDS was used to enrol women into two cohorts to estimate the impact of DREAMS on HIV incidence among YWSS after two years of follow‐up 18. The two DREAMS sites were the largest cities in Zimbabwe; the non‐DREAMS sites smaller urban towns. In all sites, the national HIV prevention and treatment programme for FSW, “Sisters with a Voice,” provides support and services, including HIV testing, community mobilization activities and condoms 10. Details of the impact evaluation, including site selection, are published elsewhere 18.

### 2.2 Study population

YWSS were eligible if they were aged 18 to 24, had sold sex (defined as sex in exchange for money and/or material support, and that the sex act would not have happened in the absence of an exchange) in the past month, and were not planning to move from the site within the next six months. Our definition of YWSS thus included women who did not self‐identify as FSW, but who considered themselves involved in sex work.

### 2.3 Data collection

Similar to other countries in the region, sex work in Zimbabwe predominantly occurs in bars and on the street, with FSW being well‐networked, and brothels uncommon 19, 20. As such, and in the absence of a sampling frame, RDS is an appropriate method to recruit YWSS, assuming they are as well‐networked as older FSW 20. The RDS surveys used identical procedures across the six sites. In 2017, community mapping was conducted in each site to identify geographical locations where women sell sex and the socio‐demographic characteristics of YWSS 21. Mapping comprised informal discussions with outreach workers, young women, YWSS, members of the community and observations at locations where young women meet sexual partners. Details of the mapping process and RDS procedures are available elsewhere 18, 21.

Through community mapping, “seed participants” representative of the population of YWSS in each study site were invited to initiate RDS. Six seed participants were recruited in each of the four comparison sites and 10 in each of the two DREAMS sites. The number of seed participants aimed to achieve sample sizes of 1200 women in the non‐DREAMS site and 1200 women in the DREAMS site, to have power to detect any impact of DREAMS at follow‐up 18.

At survey launch, seeds were interviewed in a central location and given two coupons to recruit two further young women aged 18 to 24 years, whom they knew and who sold sex 18. Upon completing survey procedures, participants were compensated USD$3 and given two coupons to continue the recruitment chain and informed that they would be compensated USD$2 for each young woman recruited to the study. Checks were in place to minimize repeat participation and assess that coupons were genuine, and young women were assessed for study eligibility 18. The recruitment process was continued over six waves in each site to reach the sample size required for the impact evaluation and achieve equilibrium across characteristics of YWSS 18.

YWSS completed an interviewer‐administered questionnaire and were offered HIV testing. The questionnaire was adapted from previous FSW surveys 22, and included questions on demographics, sexual behaviours, the locally validated Shona Symptom Questionnaire to assess risk of common mental health disorders (CMD) 23, alcohol use, experience of violence, and awareness and use of health services. To know whether women self‐identified as FSW, they were asked: “Do you consider yourself to be a sex worker?” Women were also asked details of their three most recent sexual partners, including condom use at last sex, and condom‐less sex in the previous month. We also collected data to determine personal network size for RDS estimation, asking women the number of YWSS whose name they knew, who they had seen in the previous four weeks, and whom the participant would consider recruiting to the study. HIV testing was performed by a nurse using rapid HIV tests on a finger‐prick blood sample according to national guidelines 24, with test results returned to the women.

### 2.4 Key variables

Key variables were demographic and socio‐economic characteristics, sexual behaviours and use of health services, whether women self‐identified as FSW or not (non‐identifying‐YWSS), and HIV infection. We focused on factors hypothesized to be associated with acquiring HIV that DREAMS could address, including educational attainment, marital status, whether women ever felt unable to decline sex because of the financial/material support offered in exchange for sex, women's reported number of clients, whether women self‐identified as FSW, risk of CMD, condom use and experience of physical violence 17. Although not modifiable by DREAMS among recruited women, we also explored whether age at first selling sex and years’ duration of selling sex were associated with HIV to better understand risk among this population.

### 2.5 Analysis strategy

**Figure 1:**
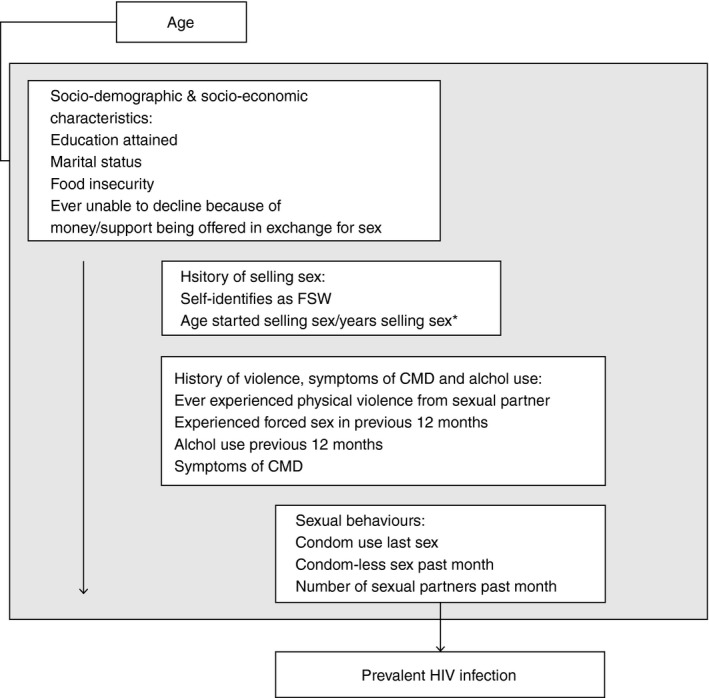
Conceptual framework for exploring the association between variables of interest and prevalent HIV infection.CMD, common health disorder; *Not adjusted for each other and not included in adjusted model for sexual behaviors. Variables in boxes below a given variable of interest were not included in models to obtain the adjusted association between that variable and prevent HIV infection.

**Figure 2:**
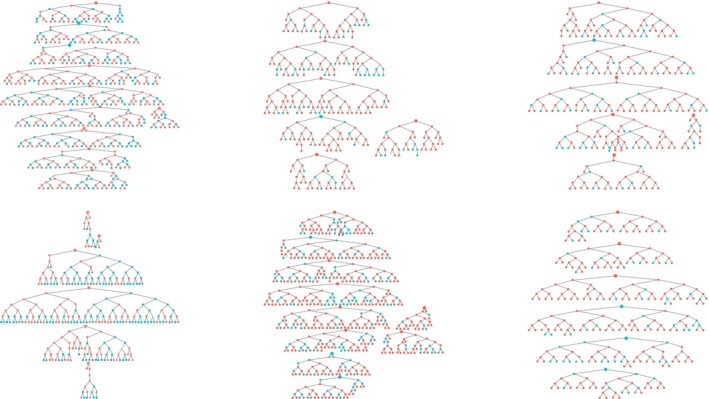
Recruitment trees. Blue circles represent women who do not identify themselves as FWS red circles represent women who identify themselves as FWS. The larger circles denote speed participants.

**Figure 3:**
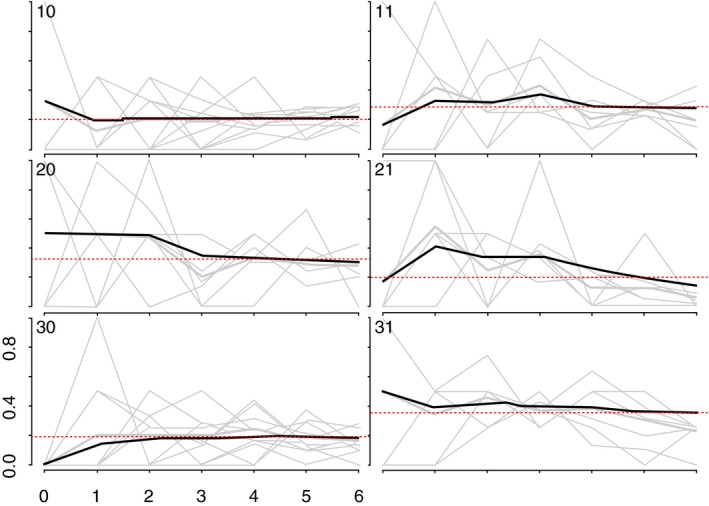
Convergence of the proportion of HIV positive YWSS. The heavy black ines indicate the cumulative RDS‐II weighted estimate overall for each site, while the grey lines are unweighted proportions for each seed, by sample wave.

**Figure 4:**
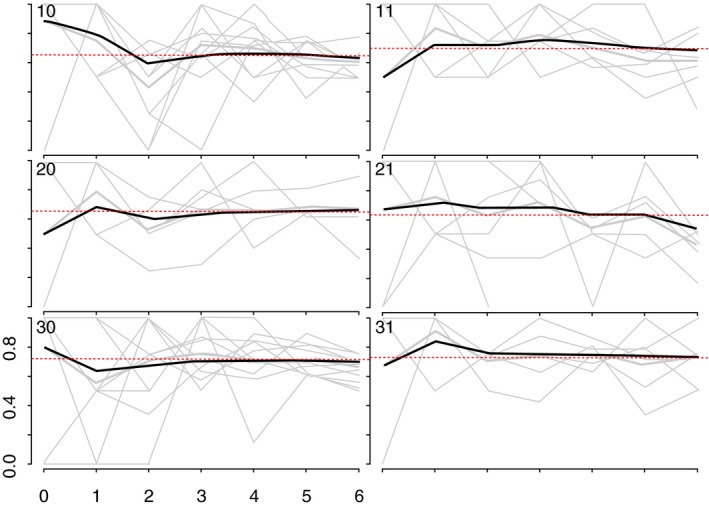
Convergence of the proportion of YWSS who reported to be aged 20 to 24 years. The heavy black lines indicate the cumulative RDS‐II weighted estimate overall for each site, while the grey lines are unweighted proportions for each seed, by sample wave.

We generated recruitment trees to visualize recruitment across the sites 25. As more individuals are recruited through RDS, the sample should become more representative of the target population 25, 26. To assess this, we used combined convergence and bottleneck plots to visualize whether the proportion of selected outcomes appeared to stabilize over recruitment 25. The variables plotted were: HIV prevalence, age at time of the survey, whether women self‐identified as FSW, and years’ selling sex (Appendix 1; Figures 1, 2, 3, 4).

Data were weighted using “RDS‐II” (weighting women by the inverse degree of the reported size of their YWSS network, that is the number of women that they could have recruited to the survey) 27 and weights normalized by site. Seed participants were excluded from all analyses. We calculated the RDS‐II‐weighted prevalence of characteristics, sexual behaviours and services use, including the site‐level ranges of these factors, and explored whether they differed by whether women self‐identified as FSW or not, adjusting for women's age and recruitment site.

We used weighted logistic regression analyses to estimate the odds ratio of prevalent HIV infection by variables of interest. In adjusted analyses, we used a distal‐proximal framework to avoid over‐adjusting variables for those that might mediate their effect on HIV 28. As such, we ran separate regression models with a different set of covariates for each variable, to avoid adjusting for covariates that may act as mediators. For example, in estimating an adjusted association between education and prevalent HIV infection, we did not adjust for sexual behaviours hypothesized to lie on any causal pathway between education on HIV (Figure 1). All models were adjusted *a priori* for age and study site. In descriptive analyses, education was categorized as: no/incomplete primary, complete primary, completion of some or all secondary education (Form 1‐3 and Form 4‐6), college/certification/degree, or whether women were still in school. Marital status was categorized as single/never married, currently married/cohabiting, divorced or widowed. In regression analyses, due to small numbers, complete secondary was combined with college/certification/degree, and divorced/widowed combined (ever‐married).

We hypothesized that whether women identified as FSW would modify the effect of factors found to be associated with prevalent HIV infection and explored whether there was statistical evidence for effect modification by self‐identification. Where there was evidence that identifying as FSW modified the association between factors and HIV infection in unadjusted analyses (*p *< 0.1) we further explored evidence for effect modification in adjusted analysis.

### 2.6 Ethics

The Ethics Committee of the London School of Hygiene and Tropical Medicine (Ref 11835) and the Medical Research Council of Zimbabwe (Ref MRCZ/A/2085) approved the DREAMS impact evaluation. All women were given information about the study and asked for written informed consent to participate.

## 3 Results

**Figure 5:**
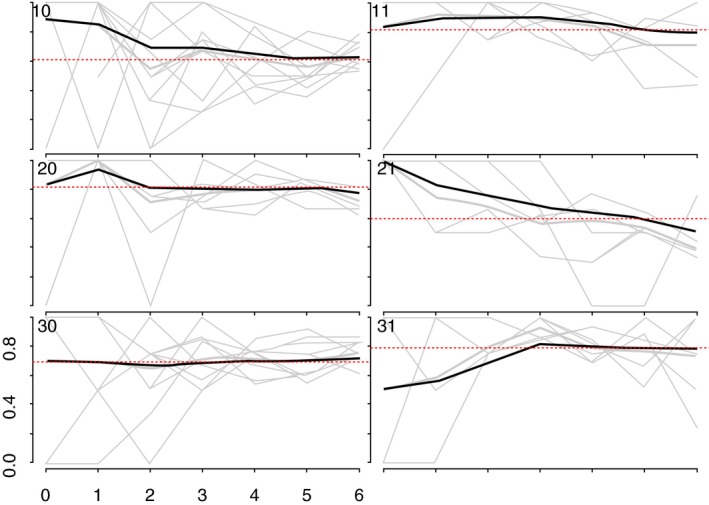
Convergence of the proportion of YWSS who self‐identify as FSW. The heavy black lines indicate the cumulative RDS‐II weighted estimate overall for each site, while the grey lines are unweighted proportions for each seed, by sample way.

**Figure 6:**
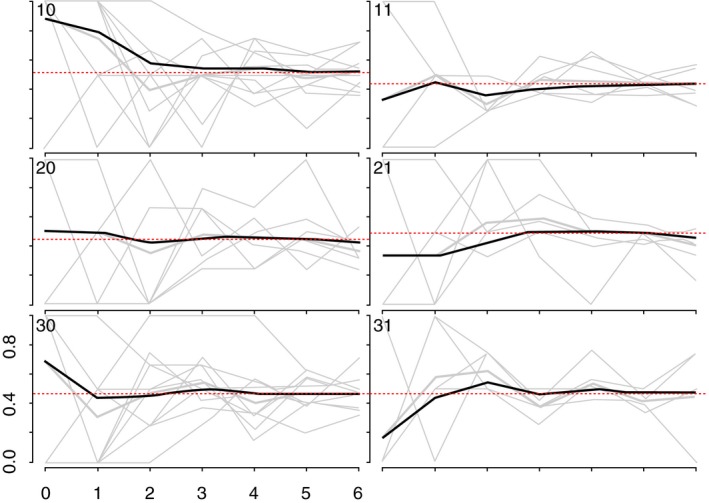
Convergence of the proportion of YWSS who reported 3 or more years of selling sex. The heavy black lines indicate the cumulative RDS‐II weighted estimate overall for each site, while the grey lines are unweighted proportions for each seed, by sample wave.

We recruited 44 seeds across six study sites. One seed did not initiate a recruitment chain and was replaced. The mean age of the seeds was 20.6 years, and most (71.4%) identified as FSW. One‐third of seeds (33.1%) tested HIV‐positive. Across the six sites, the 44 seeds recruited 2387 women over six waves (Figure 2). HIV prevalence and age appeared to stabilize in all but one site, suggesting our sample was representative of HIV prevalence and age distribution of YWSS in five of the six sites (Figures 3 and 4). For the proportion of women recruited who self‐identified as FSW, equilibrium was reached in five sites suggesting the sample is representative of the proportion of YWSS who identify as FSW in all but one study site (Figure 5). The proportion reporting ≥3 years of selling sex stabilized in all sites (Figure 6).

### 3.1 Characteristics of women

Over half of the YWSS were single or never married, 41.6% completed Form 1‐3 of secondary school, and over half supported at least one child aged <18 years (Table 1). Forty‐one percent (40.5%) started selling sex aged 15 to 17, and most (76.9%) reported that selling sex was the main way they supported themselves. Insufficient food in the previous month was reported by 54.6% of women and 33.7% reported symptoms of CMD. Fifty‐six percent of women reported selling sex to ≥4 men (clients) in the past month.

**Table 1:** Characteristics of women recruited to the impact evaluation of DREAMS, overall (N = 2387) and by whether they do not (N = 730) or do identify as a FSW (N = 1637), Zimbabwe, 2017

	All women (N = 2387)^a^		YWSS not identifying as FSW (N = 730; RDS %: 32.7%)		YWSS identifying as FSW (N = 1637; RDS %: 67.3%)		*p*‐value
	Raw n	RDS‐ Adjusted % (site‐level range)	Raw n	RDS‐Adjusted %	Raw n	RDS‐ Adjusted %	
HIV prevalence^b^ (6 and 10 missing data respectively)	543	23.6 (13.9 to 34.2)	113	**14.8**	429	**28.1**	**0.002**
Knows HIV‐positive status among women testing HIV‐positive (6 FSW missing data)	299	54.9 (50.0 to 62.5)	53	43.5	246	57.8	0.12
Age							
18	448	20.9 (15.3 to 28.5)	202	**30.8**	240	**15.8**	**<0.001**
19	371	15.8 (11.9 to 18.0)	142	**19.5**	225	**14.0**	
20	267	10.7 (8.0 to 13.2)	102	**12.9**	161	**9.5**	
21	291	11.3 (9.6 to 15.1)	87	**11.7**	203	**11.2**	
22	374	15.2 (11.3 to 19.3)	79	**10.0**	294	**17.9**	
23	471	19.6 (12.6 to 26.6)	87	**11.4**	380	**23.7**	
24	165	6.5 (3.6 to 7.9)	31	**3.7**	134	**7.9**	
Marital status							
Single/never married	1397	57.5 (42.5 to 83.2)	529	**72.9**	850	**49.7**	**<0.001**
Currently married/cohabiting	49	2.3 (0.6 to 4.0)	25	**3.4**	24	**1.8**	
Divorced	918	39.3 (12.7 to 55.4)	175	**23.6**	741	**47.3**	
Widowed	23	0.9 (0 to 2.9)	1	**0.1**	22	**1.3**	
Number of children <18‐years old that she supports (1 FSW missing data)							
0	800	33.7 (25.6 to 43.3)	314	**43.8**	478	**28.6**	**0.02**
1 to 2	1313	54.8 (48.9 to 68.1)	344	**45.4**	959	**59.5**	
3	273	11.6 (4.1 to 20.8)	72	**10.8**	199	**11.9**	
Education level or attainment (if not still in school)							
None/incomplete primary	171	8.7 (2.0 to 17.3)	30	**4.9**	**141**	**10.7**	**<0.001**
Complete primary	218	9.9 (5.9 to 12.9)	50	**7.6**	**166**	**11.1**	
Form 1‐3	975	41.6 (33.8 to 45.9)	217	**30.7**	**753**	**47.0**	
Form 4‐6	855	33.3 (25.3 to 45.2)	328	**43.0**	**516**	**28.4**	
College/certificate or degree	13	0.4 (0 to 0.8)	8	**0.7**	**5**	**0.2**	
In school	155	6.1 (1.4 to 9.8)	97	**13.2**	**56**	**2.7**	
Insufficient food in past month^c^							
No	1058	45.4	361	**50.9**	690	**42.8**	**0.02**
Yes	1329	54.6 (45.8 to 66.0)	369	**49.1**	947	**57.2**	
History of selling sex							
Age started selling sex (3 YWSS not identifying as FSW missing data)							
10 to 14	94	4.0 (3.1 to 5.0)	21	**2.9**	73	**4.6**	**0.001**
15 to 17	972	40.5 (31.7 to 51.5)	333	**45.9**	628	**37.6**	
18 to 19	721	29.9 (27.7 to 32.2)	231	**32.0**	483	**29.0**	
20 to 24	597	25.6 (13.6 to 33.3)	142	**19.2**	453	**28.9**	
Duration selling sex (in years) (3 YWSS not identifying as FSW missing data)							
<2	724	32.9 (26.9 to 35.3)	274	**40.8**	445	**29.1**	**0.003**
2 to 3	967	39.8 (33.9 to 44.9)	308	**40.6**	647	**39.1**	
4 to 5	420	17.7 (15.3 to 19.7)	95	**12.9**	322	**20.1**	
6^c^	273	9.7 (8.3 to 13.5)	50	**5.7**	223	**11.8**	
Selling sex is main support (1 YWSS not identifying as FSW missing data)							
No	487	23.1	301	**46.0**	181	**11.9**	**<0.001**
Yes	1896	76.9 (70.9 to 88.4)	428	**54.0**	1456	**88.1**	
Ever unable to decline sex due to money/material support offered (7 YWSS not identifying as FSW missing data)							
No	733	33.3	269	**42.6**	461	**29.0**	**<0.001**
Yes	1647	66.7 (62.8 to 75.6)	454	**57.4**	1176	**71.0**	
Alcohol consumption of risk of CMD							
Frequency of alcohol consumption in previous 12 months							
Never	1057	50.9 (25.2 to 62.6)	408	**64.1**	641	**44.6**	**<0.001**
Once a month or less	345	14.7 (11.6 to 19.1)	123	**16.8**	216	**13.5**	
2 to 4 times a month	325	12.2 (7.8 to 18.9)	74	**7.4**	248	**14.4**	
2 to 3 times a week	366	12.4 (7.9 to 27.2)	83	**7.9**	282	**14.7**	
4 or more times a week	294	9.8 (8.3 to 15.9)	42	**3.8**	250	**12.8**	
At risk of a common mental health disorders^d^							
No	1540	66.3	510	**72.4**	1021	**63.4**	**0.003**
Yes	847	33.7 (24.4 to 39.5)	220	**27.6**	616	**36.6**	
Experience of physical and sexual violence							
Ever experienced physical violence from a partner							
No	1415	61.2	502	**69.6**	904	**57.1**	**0.001**
Yes	972	38.8 (35.4 to 44.9)	228	**30.4**	733	**42.9**	
Experienced sexual violence past 12 months (1 YWSS who did not identify as FSW missing data)							
No	1996	84.2	631	**87.4**	1347	**82.5**	**0.03**
Yes	390	15.8 (12.5 to 20.4)	98	**12.6**	290	**17.5**	
Recent sexual behaviours							
Number of partners sold sex to past month (1 and 14 missing data respectively)							
1 to 3	965	44.2 (27.4 to 72.3)	518	**76.1**	434	**28.2**	**<0.001**
4 to 9	662	26.2 (19.7 to 33.8)	128	**14.5**	529	**32.0**	
10	745	29.6 (8.0 to 44.6)	83	**9.4**	660	**39.8**	
Condom used last sex all recent three partners (16 and 6 missing data respectively)							
No	807	35.5	268	38.1	530	34.0	0.56
Yes	1558	64.5 (52.3 to 75.5)	446	61.9	1101	66.0	
Any condom‐less sex with recent partners in the previous month (38 and 10 missing data respectively)							
No	1312	54.9	389	56.5	916	54.5	0.10
Yes	1027	45.1 (36.8 to 55.4)	303	43.5	711	45.5	
Access to HIV and related services							
Heard of Sisters clinic (1 and 1 missing data respectively)							
No	1485	66.7	511	**75.5**	961	**62.4**	**0.001**
Yes	900	33.3 (7.0 to 50.3)	218	**24.5**	675	**37.6**	
Ever been to Sisters clinic (1 and 1 missing data respectively)							
No	1807	78.5	628	**88.7**	1163	**73.4**	**<0.001**
Yes	578	21.5 (4.0 to 32.0)	101	**11.3**	473	**26.6**	
Heard of PSI New Start Centre (1 and 1 missing data respectively)							
No	961	48.1	298	50.5	658	47.2	0.63
Yes	1424	51.9 (34.9 to 77.6)	431	49.5	978	52.8	
Ever been to PSI New Start Centre (1 and 1 missing data respectively)							
No	1654	75.8	531	80.3	1111	73.8	0.34
Yes	731	24.2 (11.0 to 44.9)	198	19.7	525	26.2	
Tested within last six months (4 and 10 missing data respectively)							
No	748	33.3	278	**40.3**	461	**29.9**	**0.001**
Yes	1625	66.6 (65.7 to 70.6)	448	**59.7**	1166	**70.1**	
Heard of PrEP among women not self‐reporting their HIV‐positive status (13 and 46 missing data respectively)							
No	1588	79.3	503	80.9	1070	78.4	0.26
Yes	646	20.7 (5.2 to 52.6)	196	19.1	445	21.6	
Accessed condoms for free last time accessed condoms (37 and 18 missing respectively)							
No	540	22.4	195	27.1	345	20.2	0.41
Yes	1772	77.6 (62.9 to 91.6)	498	72.9	1274	79.8	
Accessed services last time had STI symptoms (among reporting symptoms; N = 624, 1 and 2 women missing data respectively)							
No	191	33.7	58	45.8	131	30.5	0.13
Yes	430	66.3 (42.4 to 79.7)	77	54.2	349	69.5	

Weighted percent in bold are variables where there was statistical evidence for an association between identifying a FSW after controlling for age and site; *p*‐values are from wald tests. FSW, female sex workers; PrEP, pre‐exposure prophylaxis; PSI, Population Services International; RDS, respondent‐driven sampling; STI, sexually transmitted infection; YWSS, young women who sell sex.

a 20 women missing data on whether or not they identify as FSW

b HIV‐test results missing for 18 women

c defined as responding Yes to any one of three questions from Household Food Insecurity Access Scale 29

d Threshold for experiencing CMD (common mental health disorders) was at 8.

Sixty‐seven percent (67.3%) of women self‐identified as FSW. Adjusting for age and site, FSW and non‐identifying YWSS differed. FSW were older, and by age, had lower levels of education, were more likely to be divorced/widowed and reported higher numbers of clients in the previous month (Table 1). A higher proportion of FSW reported that selling sex was the primary means by which they supported themselves (88.1% vs. 54.0% respectively), and FSW reported selling sex for more years, with 70.9% of FSW and 59.2% of non‐identifying YWSS reporting selling sex for >2 years (Table 1).

Use of HIV‐related services was generally higher among FSW; a higher proportion of FSW had heard of and ever attended a Sisters clinic, had sought treatment when they last experienced symptoms of a sexually‐transmitted infection and knew their HIV‐positive status (Table 1). More FSW reported an HIV test within the previous six months (70.1% vs. 59.7% respectively).

### 3.2 HIV prevalence and factors associated with prevalent HIV

HIV prevalence was 23.6% overall: 28.1% among FSW and 14.8% among non‐identifying YWSS (Table 1). HIV prevalence was higher among women reporting no/incomplete primary education (40.0%) relative to women with incomplete secondary education (Form 1‐3; 25.2%. adjOR = 1.79 95%CI 1.16, 2.75; *p* < 0.001). Controlling for age and education, HIV prevalence was higher among FSW than non‐identifying YWSS (adjOR = 1.43 95%CI 1.04, 1.95; *p* = 0.03). Women who started selling sex aged 10 to 14 had a higher prevalence of HIV (33.7%) relative to women who started selling sex aged 20 to 24 (29.4%; adjOR: 2.51 95%CI 1.42, 4.43; *p* = 0.004).

We found little evidence that the association between variables explored and HIV infection were modified by whether women self‐identified as FSW. There was, however, strong evidence that identification as FSW modified the association between number of clients in the previous month and HIV infection (*p*‐value for interaction = 0.02). Among women who did not identify as FSW, reporting 10+ clients was associated with higher prevalence of HIV relative to women reporting 1 to 3 (41.2% vs. 10.9%). Although there was little evidence of an association among FSW (35.4% vs. 21.6%; Table 2), the trend was similar, with prevalence of HIV infection increasing with reported number of clients.

**Table 2:** HIV prevalence and factors associated with HIV among non‐seed participants recruited through respondent driven sampling, Zimbabwe 2017 (N = 2387)

Characteristics		HIV prevalence (raw n)	RDS‐weighted HIV prevalence (%)	Age‐ and site‐adjusted OR	Fully adjusted OR^a^
Age at time of survey (years)	18	52	10.6		1.0
	19	41	13.5		1.36 (1.27, 1.46)
(adjOR is for every year increase in	20	48	20.1		
age)	21	66	23.3		
	22	106	30.8		
	23	158	32.7		
*p*‐value	24	72	52.1		<0.001
Education	No education/primary incomplete	68	40.0	1.79 (1.16, 2.75)	1.79 (1.16, 2.75)
	Complete primary	67	31.1	1.07 (0.69, 1.64)	1.07 (0.69, 1.64)
	Form 1‐3	268	25.2	1.0	1.0
	Form 4‐6 or higher	140	15.6	0.55 (0.40, 0.75)	0.55 (0.40, 0.75)
*p*‐value					<0.001
Married	Single/never married	242	18.2	1.0	
	Currently married	12	26.1	1.21 (0.47, 3.11)	–
	Divorced/widowed/separated	289	31.2	1.34 (1.01, 1.80)	–
Insufficient food in previous month^b^	No	239	23.3	1.0	–
	Yes	304	23.8	1.03 (0.79, 1.33)	–
Ever unable to decline sex due to cash/material support offered in exchange	No	161	22.6	1.0	–
	Yes	382	24.3	1.01 (0.77, 1.33)	–
History of Selling Sex					
Self‐identifies as FSW^c^	No	113	14.8	1.0	1.0
	Yes	429	28.1	2.04 (1.52, 2.74)	1.43 (1.04, 1.95)
*p*‐value					0.03
Age started selling sex^d^	10 to 14	30	33.7	3.41 (1.90, 6.13)	2.51 (1.42, 4.43)
	15 to 17	187	19.9	1.95 (1.35, 2.83)	1.76 (1.21, 2.57)
	18 to 19	152	22.6	1.39 (0.99, 1.95)	1.37 (0.97, 1.92)
	20 to 24	174	29.4	1.0	1.0
*p*‐value					0.004
Number of years reported selling sex^d^	<2	141	19.3	1.02 (0.74, 1.41)	1.05 (0.75, 1.46)
	2 to 3	179	19.8	1.0	1.0
	4 to 5	122	31.4	1.46 (1.03, 2.06)	1.38 (0.97, 1.96)
	6^e^	101	40.1	1.80 (1.20, 2.70)	1.48 (1.00, 2.21)
*p*‐value					0.12
Experience of violence, alcohol use and symptoms of CMD					
Experienced forced sex previous 12mths^f^	No	447	23.1	1.0	–
	Yes	96	26.6	1.20 (0.86, 1.68)	–
Ever experienced physical violence from sexual partner	No	273	20.0	1.0	1.0
	Yes	270	29.2	1.44 (1.12, 1.86)	1.37 (1.05, 1.78)
*p*‐value					0.02
Symptoms of CMD	No	337	22.6	1.0	–
	Yes	206	25.7	1.18 (0.91, 1.52)	–
Frequency of alcohol consumption in previous 12 months	Never	221	21.5	1.0	–
	Once a month or less	76	23.0	1.19 (0.80, 1.77)	–
	2 to 4 times a month	75	25.7	1.35 (0.91, 1.99)	–
	2 to 3 times a week	95	27.3	1.43 (0.97, 2.12)	–
	4 or more times a week	76	28.2	1.42 (0.97, 2.08)	–
*p*‐value					
Recent sexual behaviours					
Number partners sold sex to past month^g^	1 to 3	144	15.3	1.0	1.0
	4 to 9	147	22.4	1.32 (0.95, 1.84)	1.12 (0.79, 1.59)
	10^e^	243	36.0	2.19 (1.59, 3.01)	1.68 (1.18, 2.40)
*p*‐value					0.008 (0.02)
Condom used last sex all partners^h^	No	174	21.8	1.0	–
	Yes	367	24.9	1.19 (0.91, 1.57)	–
Condomless sex last month^i^	No	308	24.7	1.0	–
	Yes	225	22.1	0.88 (0.68, 1.14)	–

Stratified analysis					
Number of men women report having sex with in exchange for money/other support			HIV+ (weighted %)	Stratum specific OR	Stratum specific adjOR
Does not identify as FSW	1 to 3	62	10.9	1.0	1.0
	4 to 9	24	17.7	1.64 (0.87, 3.07)	1.56 (0.85, 2.87)
	10^e^	27	41.2	4.90 (2.39, 10.05)	4.04 (1.97, 8.27)
*p*‐value					0.001
Identifies as FSW	1 to 3	82	21.6	1.0	1.0
	4 to 9	122	23.6	0.99 (0.66, 1.47)	0.94 (0.62, 1.41)
	10^e^	216	35.4	1.51 (1.03, 2.20)	1.31 (0.88, 1.95)
*p*‐value					0.14

*p*‐value is from Wald test. CMD, common mental health disorders; FSW, female sex worker; OR, odds ratio; RDS, respondent‐driven sampling.

a Variables included in the model for each factor were guided by the conceptual framework (Figure 1)

b defined as responding Yes to any one of three questions about whether anyone in the household slept hungry, or went a day or night without food in the past four weeks (questions from Household Food Insecurity Access Scale 29)

c 20 missing data

d 3 missing data

e 7 missing data

f 1 missing data

g 15 missing data

h 12 missing data

i 12 missing data.

## 4 Discussion

Women recruited to our study had a high prevalence of HIV infection, rising sharply with age, and increasing with lower educational attainment and number of clients. Participants were socioeconomically vulnerable, and many relied on sex work to support themselves. Non‐identifying‐YWSS differed from FSW: FSW were older, reported more clients and were more engaged with services. YWSS who may be transitioning into sex work, or who do not perceive their sale of sex to be sex work, are also at high risk of HIV and perhaps more vulnerable due to poorer engagement with services for FSW. They may benefit from less explicitly targeted services, such as those offered through the DREAMS Partnership. Such comprehensive HIV prevention and social protection programmes need, however, to be cognizant of YWSS as a group in particular need of being reached.

YWSS who do and do not self‐identify as FSW were found to differ in important ways; understanding whether or not YWSS identify as FSW is an important foundation for delivering programming acceptable to all YWSS. This dichotomy, however, provides little insight into nuanced motivations for selling sex or changes in women's identification with sex work overtime 13. The literature on transactional sex shows a continuum between transactional sex and sex work 13, 14. As described, transactional sex has been associated with agency and power; sex work with poverty, dependency and moral judgement 13, 14. The distinction between transactional sex and sex work can be vague 14, with distinctions between sex work among women who identify as FSW or not likely more vague and highly nuanced. Half the non‐identifying‐YWSS recruited to our study relied on selling sex to support themselves and had experienced a time when they were unable to decline sex because of the support offered, suggesting that, with time, these women may come to identify as FSW. Whether and how women's identification with sex work changes overtime, the contextual factors that shape women's transition into and out of formalized sex work, and the implications these transitions have on need for and access to HIV prevention and related services should be explored if programmes are to provide services appropriate to the needs of all YWSS throughout their lifecourse 14, 21.

Non‐identifying‐YWSS in our study also differed from other adolescent girls and young women in Zimbabwe, reporting higher numbers of sexual partners. In the 2015 Demographic and Health Survey, 0.7% of women aged 18 to 19 and 2.0% of women aged 20 to 24 reported 2+ partners in the previous 12‐months 30 and their age‐specific HIV prevalence was half that of these YWSS 31. The factors associated with HIV in our study were similar among FSW and non‐identifying‐YWSS, underscoring the high risk of HIV among young women involved in sex work but who do not identify as FSW. Programmes aiming to prevent HIV should target these shared determinants of risk regardless of how women identify, including through an offer of PrEP, access to HIV‐testing services, and support in condom use and negotiation skills. Yet, how these programmes are delivered should consider how women identify, with programmes not promoted as services for FSW but as non‐stigmatising services for the health and well‐being of adolescents and young people. Fear of disclosing engagement in selling sex to family/friends and healthcare workers, compounded by age and competition with older FSW, likely limits use of available services by YWSS 8, 21.

Our study is unique in that we used RDS to recruit YWSS, which proved successful to casting a “wide net” and reaching self‐identifying FSW and other women involved in selling sex. To reach all YWSS, programmes should consider network‐based approaches and use of incentivized referral vouchers, with feasibility of this approach among non‐identifying ‐YWSS demonstrated by this study. Trained peer educators should also be engaged, as they may reach younger FSW 32, 33, 34, and may prove important to retain YWSS in programmes and develop social capital among non‐identifying‐YWSS, which has been associated with increased condom use 35.

Studies in Thailand, India and Kenya found that younger women and women who recently transitioned into sex work were at high HIV risk 3, 36, 37, 38, 39. Studies from Canada, India and Nepal, show a high risk of HIV among women who started selling sex aged <18 40, 41, 42 Our study adds to this evidence, suggesting an increased HIV risk among women in Zimbabwe who start selling sex at younger ages. This increased risk may be related to vulnerabilities that push women to sell sex 1, 43, including household poverty or death of an HIV‐positive partner or parent, which are compounded by age through an influence on agency, resources, ability to negotiate condom use and physiological vulnerability of the genital tract 43, 44 By reaching YWSS at younger ages, programmes could support building young women's resilience and provide more livelihood options to prevent YWSS from transitioning into long‐term sex work 45.

## 5 Strengths and limitations

We used the same methods and an experienced team to implement RDS in all sites. Due to the nature of RDS, we cannot assess whether women recruited into the study are representative of all YWSS or whether participation was biased by how women identified. HIV prevalence and the characteristics of YWSS appeared to converge over waves in all but one of the smaller towns. In this site, bias may have been introduced by seed selection 25, and our study population may be slightly biased towards older women identifying as FSW. Community mapping in the study sites identified tertiary institutions as locations where students sold sex 21. Few women in this study reported being enrolled in college, the seed participant that was replaced was a university student and the site where HIV prevalence and characteristics did not converge has a tertiary education institution. RDS may be less effective among these YWSS 21, or our strategy of recruiting through public clinics in some sites may have limited our reach to these women, who are more likely to be middle class and perhaps unwilling to access these locations. Implementing RDS that combined reaching FSW and non‐identifying‐FSW and was led by an organization known to deliver the Sisters with a Voice FSW programme may have had implications for who we recruited. These findings provide important lessons for future applications of RDS among YWSS.

We were interested in exploring risk factors for HIV acquisition. Using HIV prevalence as the outcome limits our understanding of the causal relationship between the characteristics and behaviours considered and HIV acquisition, since infection may have preceded the behaviours measured here. There may be biased reporting; non‐identifying‐FSW, who made an active decision not to formalize their sex work, may have been less likely to accurately report number of clients than FSW. Bias in reported condom use and other sexual behaviours are well‐documented in the literature, yet we found that levels of condom use at last sex were similar to other studies among FSW and underscores the need for women to have the option to use PrEP 46, 47

## 6 Conclusions

We used RDS to recruit YWSS who identified as FSW and YWSS who did not. The YWSS who participated in our study had a high prevalence of HIV, experienced other socioeconomic vulnerabilities, and remain a key population for comprehensive HIV programmes. To reach all YWSS, programmes need to consider social network‐based approaches and address the shared determinants of HIV risk 29 Understanding the determinants underlying women's transitions into and out of sex work throughout their lifecourse, and the implications of these transitions on need for and access to services is needed to reach and retain women at high HIV risk and during periods of highest risk.

## Competing interests

None reported.

## Authors’ contributions

BH conducted the analysis and wrote the first draft. SC conducted the RDS diagnostics and contributed to writing. SF was involved in planning the analysis. TC and PM lead the data collection. JB provided critical revisions the article, particularly the discussion. SF, IB, BH, JH and FC were involved in the conception of the study, interpretation of results and critical revision of the article. All authors contributed to the writing and have read and approved the final version.
